# Dedication: Professor Dame Georgina Mace DBE FRS (1953–2020)

**DOI:** 10.1098/rstb.2024.0458

**Published:** 2025-01-09

**Authors:** Jon Bridle, Andrew Balmford, Sarah M. Durant, Richard D. Gregory, Richard Pearson, Andy Purvis

**Affiliations:** ^1^ Centre for Biodiversity and Environment Research, Department of Genetics, Evolution and Environment, University College London, London WC1E 6BT, UK; ^2^ Department of Zoology, University of Cambridge, Cambridge, UK; ^3^ Conservation Research Institute, University of Cambridge, Cambridge, UK; ^4^ Institute of Zoology, Zoological Society of London, London, UK; ^5^ RSPB Centre for Conservation Science, Sandy, Bedfordshire, UK; ^6^ Biodiversity Futures Lab, Natural History Museum, London SW7 5BD, UK; ^7^ Georgina Mace Centre for the Living Planet, Silwood Park, Ascot SL5 7PY, UK


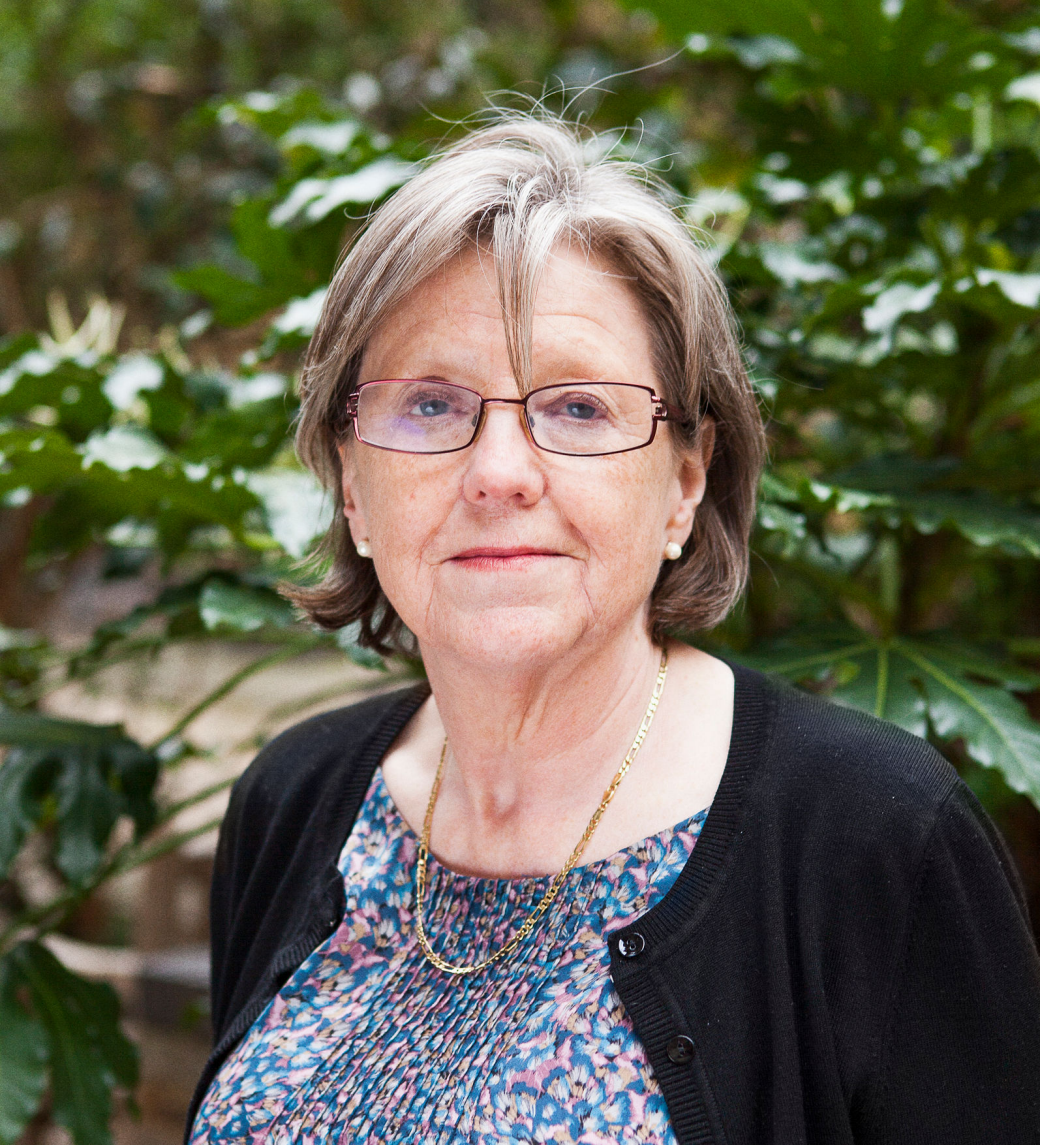


This volume of papers arose from a scientific meeting at the Royal Society, London on 12 and 13 June 2023, entitled ‘Recovering nature: building on Georgina Mace’s work to ensure a biodiverse and liveable future’. It is fitting that this collection of papers appears in *Philosophical Transactions B*, because Georgina was Editor-in-Chief of this journal from 2008 until 2010. She was the first female Editor-in-Chief of a Royal Society journal, and this collection appears in 2025, which is the 80th anniversary of the first women to be elected to the Royal Society.

Georgina was a profoundly influential scientist in biodiversity research and conservation. After completing a PhD at the University of Sussex in 1979, from 1986 to 2006 she worked at the Zoological Society of London (ZSL), becoming Director of Science from 2000 to 2006. At ZSL, she was central to the design of many new science-based indicators of species’ global status and of the trends in ecosystems now used to measure progress towards meeting international biodiversity targets. These included the Red List Index, which quantifies changes in the level of extinction risk from repeated assessments, and the Living Planet Index, which reports abundance trends across populations of more than 5000 vertebrate species. The ongoing use and influence of both of these pioneering metrics is reviewed in this issue.

Georgina then went to Imperial College London in 2006 to become the Director of the Centre for Population Biology at Silwood Park, where she helped galvanise the UK’s commitment to ending ecosystem degradation through her work on the National Ecosystem Assessment. In 2012, Georgina moved to University College London to become the founding director of a new research centre—the Centre for Biodiversity and Environment Research (CBER). At CBER, she continued to combine her fundamental interest in the processes that drive the creation and maintenance of biodiversity with measuring and analysing patterns of recent biodiversity loss, and led transdisciplinary research demonstrating and quantifying nature’s value to people. The working culture and ambition created by Georgina has allowed the centre, and those lucky to be associated with it, to thrive. It is also telling that the three research themes that Georgina defined during CBER’s foundation are still actively pursued over a decade later, with scope for their study recently expanded by the opening of CBER’s People and Nature Lab (2022) at UCL’s new East campus in Stratford.

The 2023 Royal Society Discussion meeting set out to highlight the huge influence of Georgina’s life, work and interests on biodiversity and environmental research today. It did this through contributions by some of those inspired by Georgina’s work and life. Speakers continually highlighted how they are developing themes started by Georgina and applying them to new problems and solutions, and with new techniques.

What came across very strongly during the two days shared by attendees in London, as well as those joining remotely from across the world, was how generous Georgina was with her ideas, support and time, especially towards researchers early in their careers. And how her kindness, calmness and acuity, shot through with an astute sense of humour, inspired a whole generation of researchers. Such qualities are increasingly essential for biodiversity researchers, as we bear more and more detailed witness to the widespread and rapid loss of the natural world.

We still find ourselves asking whether Georgina would find something worthwhile, or interesting, before attempting it. And we miss sharing our disappointments and failures with her as much as our successes. She saw the world as it was—imperfect and imperfectly understood—but remained keen to find leverage and effect change even without exact answers. She understood all too well that action cannot wait for perfection.

We have tried to reflect Georgina’s diverse interests in the articles contained in this issue, although given the breadth of her contributions it is inevitable that there is much left out. In particular, we have sought to highlight her deep interest in asking why the world is the way it is, as well as her passion for using that understanding to make the planet more fair and more secure, especially for the world’s disadvantaged and for future generations. A world where people like Georgina have more agency will be a world much better for most, and one that will be much more interesting to live in.

Beyond the papers in this issue, we remember Georgina’s joy and excitement when thinking about some fundamental scientific question, as well as her patience, humour and continued optimism as we and our societies continued to fall short of delivering the transformative change that science shows is needed.

Georgina championed an inspirational goal of ‘bending the curve of biodiversity loss’. That not only should we slow biodiversity loss, we must also aim for a nature-positive future, to restore a rich and biodiverse planet that can support the health, happiness and potential of current and future generations. A future we can get to safely, and one where humanity starts to live on this planet as if we intend to stay here.

## Data Availability

This article has no additional data.

